# Uncovering a suppressor effect in the relationship between psychological capital and employment expectations: a chain mediation model among vocational undergraduates

**DOI:** 10.3389/fpsyg.2025.1461983

**Published:** 2025-06-17

**Authors:** Zerui Huang, Ismi Arif Ismail, Akmar Hayati Ahmad Ghazali, Jeffrey Lawrence D'Silva, Haslinda Abdullah, Zeqing Zhang

**Affiliations:** ^1^Institute for Social Science Studies, Universiti Putra Malaysia, Serdang, Malaysia; ^2^School of Culture and Communication, Guangdong Business and Technology University, Zhaoqing, China; ^3^Faculty of Educational Studies, Universiti Putra Malaysia, Serdang, Malaysia; ^4^Faculty of Modern Languages and Communication, Universiti Putra Malaysia, Serdang, Malaysia

**Keywords:** psychological capital, employment expectations, educational flow experience, active coping style, vocational undergraduates, suppressor effect

## Abstract

**Purpose:**

This study investigates the relationship between psychological capital and employment expectations among vocational undergraduate students, with a particular focus on the mediating roles of educational flow experience and active coping style.

**Methods:**

Data were collected through a questionnaire survey administered to 693 vocational undergraduate students at a university in Guangdong Province, China. The instruments used included the Positive Psychological Capital Scale (PPS), Career Expectation Scale (CES), Educational Flow Scale (EduFlow-2), and Coping Style Scale (CSS).

**Results:**

(1) Psychological capital was found to exert a significant positive influence on employment expectations, educational flow experience, and active coping style. (2) Both educational flow experience and active coping style played significant mediating roles in a chain mediation model. Furthermore, a suppressor effect was identified in the mediation process.

**Conclusion:**

This study confirms the significant influence of psychological capital on employment expectations and elucidates its underlying mechanisms. It highlights the critical roles of educational flow and active coping in shaping students' employment outlook, offering practical insights for enhancing employment outcomes among vocational undergraduates.

## 1 Introduction

### 1.1 Foundation

In recent years, the Chinese government has placed increasing emphasis on strengthening vocational undergraduate education as a core component in building a modern higher education system. The Action Plan for Building a Leading Education Power (2024–2035) explicitly calls for the acceleration of a modern vocational education framework, the expansion of vocational undergraduate institutions, and the development of high-quality universities with distinctive characteristics and advanced educational standards. It further advocates for vertical and horizontal integration between vocational and general higher education systems (Hou, 2025). These strategic initiatives signify a transition in the role of vocational undergraduate education—from a supplementary track to a central pillar in serving national development goals, particularly in the context of strengthening China's human capital and technological competitiveness.

As of 2025, the number of vocational undergraduate institutions in China has reached 60, with the development pipeline for vocational education continuously expanding. Simultaneously, national policies have deepened industry–education integration and enhanced university–enterprise collaboration, facilitating the establishment of practice-oriented hubs that combine teaching, training, and research. These efforts have improved the practical relevance and industrial adaptability of vocational education (Tianzuo et al., 2025). Notably, policy reforms are actively dismantling credential-based discrimination by granting vocational graduates equal access to residency permits, employment opportunities, and professional advancement, thus fostering a more inclusive and equitable talent development environment (Fan et al., 2024).

Despite these positive developments, vocational undergraduate education continues to face multifaceted challenges. Social perceptions of vocational education remain biased, with deeply entrenched hierarchical views on academic credentials undermining the legitimacy and attractiveness of vocational tracks. Compared with elite academic institutions, vocational universities still lag in student recruitment quality, social prestige, and resource allocation (Wang, 2022). These structural disparities in perception often translate into employment disadvantages, placing vocational graduates at a relative disadvantage in job acquisition, career advancement, and salary prospects. Against the backdrop of an increasingly saturated labor market, the employment anxiety and real-world difficulties faced by vocational undergraduates are especially pronounced (Song and Xu, 2024). Without targeted psychological and systemic support, these students may experience diminished confidence, reduced career identity, and weakened future expectations—ultimately threatening the attractiveness and sustainability of vocational education.

In current scholarship, employment expectations are defined as individuals' subjective anticipations regarding their future employment status, career development trajectory, and potential for achievement prior to entering the labor market (Treuren and Anderson, 2010). These expectations not only shape career decision-making and development paths but also strongly correlate with psychological wellbeing, life satisfaction, and job satisfaction. As such, employment expectations serve as a vital indicator of youth confidence and motivation in career development (Kong et al., 2015).

While previous research has primarily focused on external factors—such as individual competencies, leadership qualities, expectation-reality gaps, work experience, and family capital—relatively little attention has been paid to the internal psychological mechanisms that influence employment expectations (Drewniak et al., 2020; Ayoobzadeh et al., 2024; Xu et al., 2025; Zhang et al., 2025). In particular, the formation pathways, regulatory mechanisms, and psychological foundations of employment expectations among vocational undergraduates remain underexplored. Given the ongoing diversification of China's higher education system and the rapid expansion of vocational universities, it is critical to investigate the marginalized status and psychological adaptation of vocational graduates in the labor market.

In recent years, the rapid expansion of China's higher education system has led to an unprecedented surge in the number of university graduates. By 2025, the number of higher education graduates is projected to reach a record high of 12.22 million (Li et al., 2025). While this surge reflects the acceleration of mass higher education, it has also intensified structural pressures on youth employment. Coupled with slowing economic growth and industrial restructuring, the overall absorptive capacity of the labor market has declined, leading to increased job competition (Yang, 2024). In this context, vocational undergraduates—due to factors such as limited degree recognition, suboptimal program alignment with labor market needs, and persistent social stigma—are more vulnerable during job searches.

In response to these challenges, the Chinese government has introduced a series of comprehensive measures aimed at alleviating employment pressure. These include deepening industry–education integration, optimizing academic program structures, incentivizing small and medium enterprises to recruit graduates, and expanding policy-driven employment opportunities (Huang et al., 2024). However, beyond systemic interventions, the internal development of students' psychological capacities, coping mechanisms, and career beliefs is equally vital. Psychological capital, as a form of positive psychological resource, has been shown to enhance individuals' resilience and goal persistence when confronting complex employment scenarios. Educational flow experiences and active coping styles may serve as key mediating mechanisms through which psychological capital influences employment expectations.

Accordingly, this study aims to examine how psychological capital, educational flow experience, and active coping style collectively shape vocational undergraduate students' employment expectations. Specifically, we seek to test a chain mediation model to explore the underlying psychological mechanisms and provide empirical evidence and theoretical insights for advancing vocational education reform and improving graduate employment outcomes in contemporary China.

### 1.2 Aims and hypotheses

Psychological capital, a key construct at the intersection of positive psychology and organizational behavior, was introduced by Luthans et al. (2007) to describe an individual's positive psychological state during growth and development. It encompasses four core components—self-efficacy, hope, optimism, and resilience—collectively known as the HERO model. This theoretical framework emphasizes that internal psychological resources not only enhance individuals' adaptability in the face of challenges but also contribute positively to goal attainment and problem-solving processes. In recent years, psychological capital has gained increasing attention in the field of education, particularly in relation to students' employability, career planning, and psychological adjustment (Baluku et al., 2021). Prior research has demonstrated that higher levels of psychological capital strengthen students' confidence and goal-directed behaviors, thereby improving their ability to manage employment-related stress and adapt to occupational environments (Zhang L. et al., 2024). Moreover, psychological capital has been shown to enhance students' career decision-making self-efficacy, reducing confusion and uncertainty in career selection and fostering clearer and more positive employment expectations (Zhou A. et al., 2024). Based on this, we propose the following hypothesis:

H1: Psychological capital positively predicts employment expectations among vocational undergraduate students.

Educational flow experience is rooted in the flow theory of positive psychology (Csikszentmihalyi, 1990) and refers to a psychological state of deep concentration, immersion, and enjoyment during learning activities (Huang et al., 2025). Such a state typically occurs when the challenge level of a task matches the learner's perceived competence, leading to heightened engagement and a distortion of time perception (Heutte et al., 2016). In the context of higher education, educational flow has been recognized as a critical factor in promoting students' intrinsic motivation and enhancing learning outcomes (Shi et al., 2024). Research has found that frequent experiences of flow during learning are closely associated with stronger motivation and greater academic achievement (Yang et al., 2025). Additionally, educational flow is linked to a range of psychological variables, including emotional regulation, self-efficacy, and academic satisfaction, which in turn shape students' career development and employment expectations (Yen and Lin, 2022). According to Vroom's expectancy theory, an individual's motivation is determined by their belief in the likelihood of goal attainment and the perceived value of the outcome (Vroom, 1964; Amali et al., 2023). Educational flow strengthens students' engagement in the learning process and their expectations for success, thereby enhancing their motivational levels and influencing their employment expectations. Thus, we propose:

H2: Educational flow experience mediates the relationship between psychological capital and employment expectations among vocational undergraduates.

Coping style is a key concept in psychology, referring to the cognitive and behavioral strategies individuals use to manage stress and challenges (Bondarchuk et al., 2024). These strategies are generally categorized as either active or passive coping (Falloon et al., 2023). Active coping strategies aim to address or mitigate stressors and include behaviors such as seeking support, proactive planning, and problem-solving. In contrast, passive coping strategies involve avoidance, denial, or emotional venting, which are often ineffective and may exacerbate psychological distress (Zou et al., 2024; Lu et al., 2021).

In the educational domain, studies have shown that active coping strategies enhance students' mental health and subjective wellbeing (Jiang et al., 2021). For instance, these strategies can increase self-efficacy and reduce anxiety and depression, thereby improving students' adaptability and life satisfaction (Fischer et al., 2021). Psychological capital is also recognized as a crucial resource for promoting active coping. Students with higher psychological capital are more confident and motivated when facing difficulties, making them more likely to adopt effective coping strategies, which in turn can alleviate employment anxiety and boost employment expectations (Zewude and Hercz, 2021). Accordingly, we propose:

H3: Active coping style mediates the relationship between psychological capital and employment expectations among vocational undergraduates.
H4: Educational flow experience and active coping style jointly mediate the relationship between psychological capital and employment expectations in a chain mediation model.

**Figure 1:**
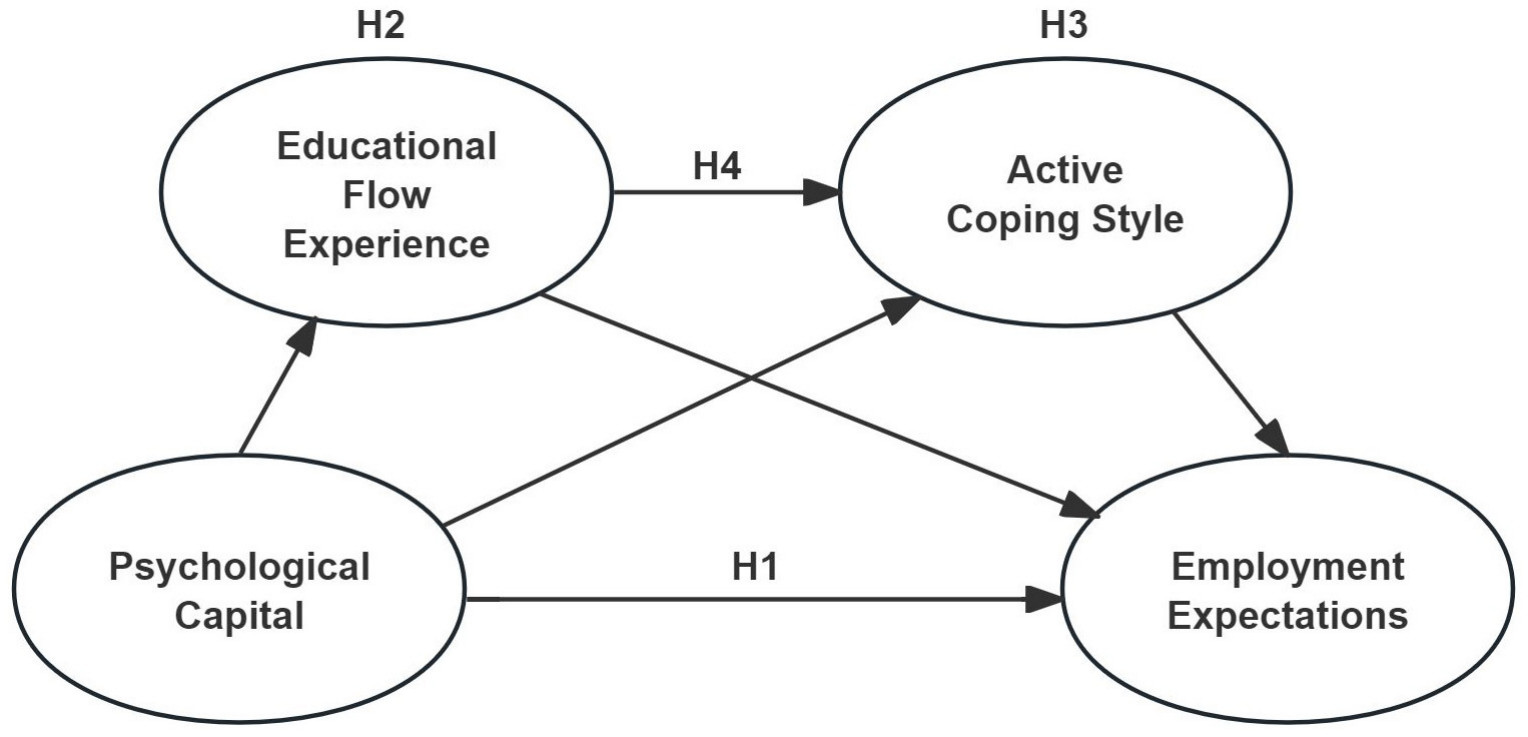
Hypothesis model.

Based on the theoretical background and literature review, a conceptual framework was developed (Figure 1), proposing that educational flow experience and active coping style serve as key mediators in the relationship between psychological capital and employment expectations, with a potential sequential pathway linking the two mediators.

## 2 Method

### 2.1 Participants

A total of 693 vocational undergraduate students from a university in Guangdong Province, China, participated in this study. Among them, 377 were female (54.40%) and 316 were male (45.60%). Their ages ranged from 18 to 22 years, with a mean age of 19.970 years (SD = 1.362). All data were collected anonymously to ensure participant privacy.

### 2.2 Instruments

#### 2.2.1 Psychological capital

The Positive Psychological Capital Scale (PPS; Zhang et al., 2010) was used to assess the four components of psychological capital. The scale includes 7 items for self-efficacy, 7 for resilience, 6 for optimism, and 6 for hope. The PPS has been validated for use among Chinese university students and exhibits strong reliability and construct validity (Pan and Wu, 2022). A 7-point Likert scale was used (1 = strongly disagree; 7 = strongly agree). Items 8, 10, 12, and 14 (resilience) and item 25 (hope) were reverse-coded. Higher scores indicated higher levels of psychological capital. Cronbach's alpha coefficients for the subscales ranged from 0.943 to 0.960. Confirmatory factor analysis yielded satisfactory fit indices: χ^2^/*df* = 2.728, RMSEA = 0.049, CFI = 0.979, NFI = 0.967, GFI = 0.917.

#### 2.2.2 Employment expectations

The Career Expectation Scale (CES; Manhardt, 1972) was used to assess students' employment expectations across four dimensions: long-term career success (6 items), work environment and interpersonal relations (5 items), autonomy and self-fulfillment (8 items), and professional values (6 items). The CES has demonstrated good psychometric properties in Chinese student populations (Chullen et al., 2015). A 7-point Likert scale was used (1 = strongly disagree; 7 = strongly agree). Higher scores indicate stronger employment expectations. Cronbach's alpha coefficients ranged from 0.853 to 0.942. Fit indices were as follows: χ^2^/*df* = 2.197, RMSEA = 0.042, CFI = 0.986, NFI = 0.975, GFI = 0.936.

#### 2.2.3 Educational flow experience

The EduFlow-2 Scale (Heutte et al., 2021) was used to measure four dimensions of educational flow: cognitive control (3 items), immersion and time transformation (3 items), loss of self-consciousness (3 items), and autotelic experience (3 items). The Chinese version has shown high reliability and validity (Shi et al., 2024). A 7-point Likert scale was used (1 = strongly disagree; 7 = strongly agree). Higher scores reflected a stronger flow experience. Cronbach's alpha values ranged from 0.884 to 0.913. Fit indices were χ^2^/*df* = 2.775, RMSEA = 0.048, CFI = 0.989, NFI = 0.983, GFI = 0.965.

#### 2.2.4 Active coping style

The Coping Style Scale (CSS; Chan, 1998) was used to assess coping styles across four dimensions: rational problem-solving (4 items), resigned distancing (4 items), seeking support and ventilation (4 items), and passive wishful thinking (4 items). The scale has demonstrated good reliability and validity in prior studies (Chan, 2007). A 7-point Likert scale was used (1 = strongly disagree; 7 = strongly agree). Items in the resigned distancing and passive wishful thinking subscales were reverse-coded. Higher scores indicated more active coping strategies. Cronbach's alpha coefficients ranged from 0.947 to 0.954. Model fit indices were χ^2^/*df* = 2.751, RMSEA = 0.049, CFI = 0.986, NFI = 0.979, GFI = 0.952.

### 2.3 Procedure

This study employed a convenience sampling method to recruit participants. Ethical approval was obtained from the Ethics Committee of Guangdong Business and Technology University on March 19, 2024 (Approval No.: 2024GS032). Data collection took place between April 10 and April 20, 2024, using an online survey platform. Before completing the questionnaire, participants were informed about the purpose and content of the study and provided written informed consent in accordance with institutional requirements. The survey was administered in a self-report format.

### 2.4 Data analysis

All data were entered, processed, and analyzed using SPSS 27.0, AMOS 27.0, and the PROCESS macro. Analytical procedures included descriptive statistics, Pearson correlation analysis, and mediation analysis to examine the chain mediation effect of educational flow experience and active coping style. To ensure consistency, all data were standardized using z-scores prior to analysis. Given the self-report nature of the data, Harman's one-factor test was employed to assess potential common method bias. Bootstrapping procedures were used to evaluate mediation effects, with 5,000 resamples and 95% confidence intervals. A mediation effect was considered statistically significant if the confidence interval for the indirect effect did not include zero.

## 3 Results

### 3.1 Common method deviation test

To assess potential common method variance, Harman's single-factor test was conducted on the measures of psychological capital, employment expectations, educational flow, and active coping. The analysis revealed 16 factors with eigenvalues >1, with the first factor accounting for only 25.93% of the total variance—well below the 40% threshold—indicating that common method bias was not a significant concern (Podsakoff and Organ, 1986).

### 3.2 Descriptive statistics

Table 1 presents the means, standard deviations, and Pearson correlations for the study variables. Psychological capital was moderately correlated with employment expectations (*r* = 0.597, *p* < 0.01), educational flow (*r* = 0.380, *p* < 0.01), and active coping (*r* = 0.449, *p* < 0.01). Employment expectations were also significantly correlated with educational flow (*r* = 0.500, *p* < 0.01) and active coping (*r* = 0.511, *p* < 0.01). Additionally, educational flow and active coping were moderately correlated (*r* = 0.349, *p* < 0.01). These results support the assumptions necessary for mediation analysis. All correlation coefficients were below 0.700, indicating no multicollinearity.

**Table 1:** Descriptive statistics and correlations of study variable.

**Variables**	**1**	**2**	**3**	**4**
1. Psychological capital	–			
2. Employment expectations	0.597^**^	–		
3. Educational flow experience	0.380^**^	0.500^**^	–	
4. Active coping style	0.449^**^	0.511^**^	0.349^**^	–
Mean	5.223	4.892	5.732	5.345
SD	0.442	0.540	0.510	0.533

*N*= 693. ^**^p < 0.01.

### 3.3 Main effect

**Figure 2:**
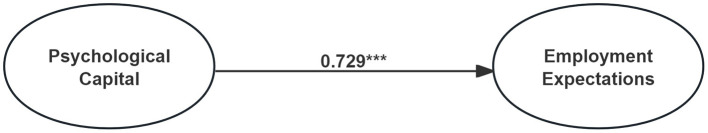
Main effect of psychological capital to employment expectation. ***p < 0.001.

When testing the main effect path using structural equation modeling (SEM), the standardized regression coefficients ranged from 0.59 to 0.86. The model demonstrated a good fit to the sample data: χ^2^ = 47.287, χ^2^/*df* = 2.489 (Schumacker and Lomax, 2004), RMSEA = 0.047 (<0.05; McDonald and Ho, 2002), CFI = 0.985, NFI = 0.975 (Hu and Bentler, 1999), GFI = 0.982, and TLI = 0.978 (Doll et al., 1994). Psychological capital accounted for 53.60% of the variance in employment expectations (β = 0.73, *p* < 0.001), supporting Hypothesis 1 (Figure 2).

### 3.4 Structural model

**Figure 3:**
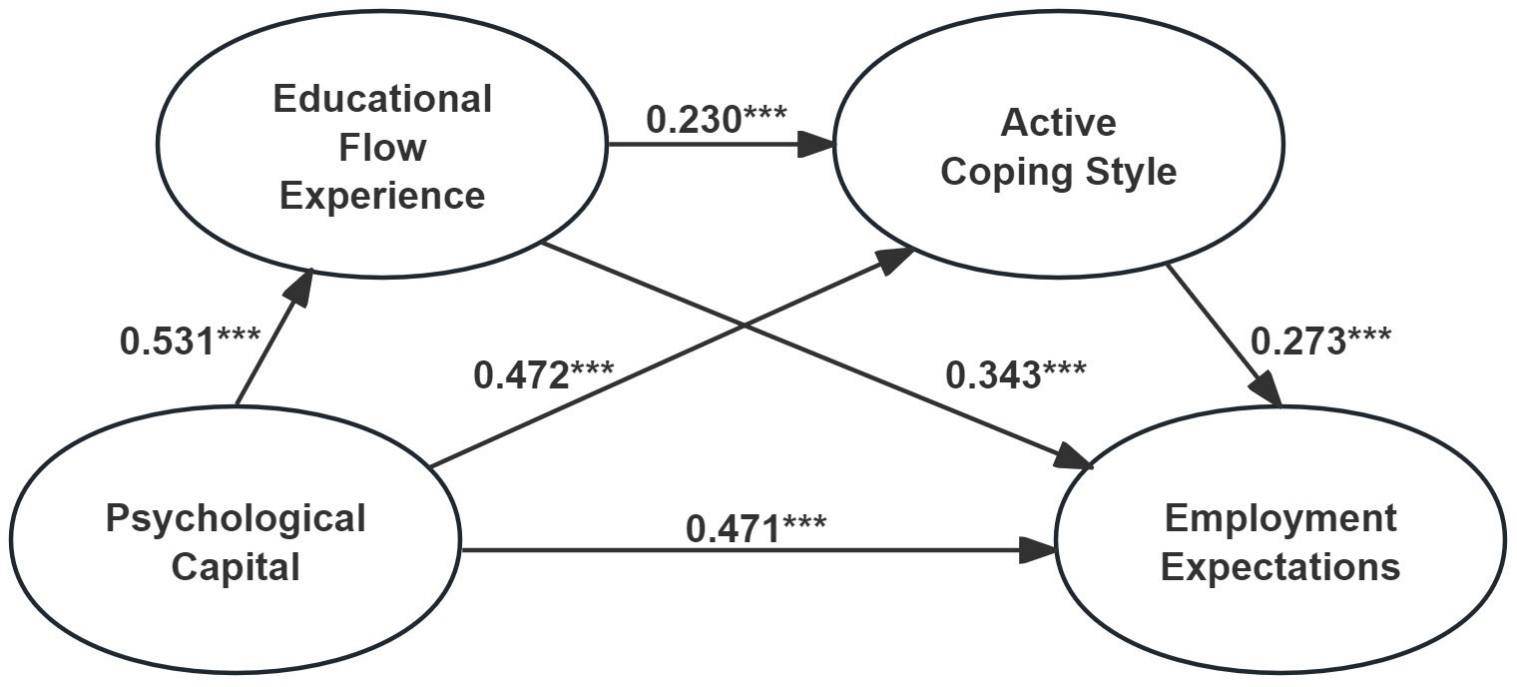
Structural model. ***p < 0.001.

In the SEM model, standardized regression coefficients for the main paths ranged from 0.230 to 0.531. The structural model exhibited acceptable fit to the data: χ^2^ = 237.556, χ^2^/*df* = 2.424 (Schumacker and Lomax, 2004), RMSEA = 0.045 (<0.05; McDonald and Ho, 2002), CFI = 0.965, NFI = 0.943 (Hu and Bentler, 1999), GFI = 0.958, and TLI = 0.958 (Doll et al., 1994). Hypotheses 2 to 4 involved the mediating variables integrated into this structural model (Figure 3).

### 3.5 Mediating effect

To test the hypotheses, the bootstrapping method was applied to examine the chain mediation model from educational flow experience to active coping style in the relationship between psychological capital and employment expectations (Hayes, 2009). As shown in Table 2, the 95% confidence intervals (5,000 resamples) did not include zero (Zhang Z. et al., 2024). The direct effect of psychological capital on employment expectations was significant (β = 0.471, *p* < 0.001). For the path “psychological capital → educational flow → employment expectations,” the 95% CI excluded zero (β = 0.124, *p* < 0.001). Similarly, for “psychological capital → active coping → employment expectations,” the 95% CI also excluded zero (β = 0.110, *p* < 0.001). For the chain path “psychological capital → educational flow → active coping → employment expectations,” the CI again excluded zero (β = 0.024, *p* < 0.001), indicating a significant chain mediation effect.

**Table 2:** Bootstrapping test result for chain mediating effects.

**Total, direct, and indirect effect**	**Effect**	**SE**	**Boot 95% CI**	
			**LLCI**	**ULCI**
PC → EFE → EE	0.124	0.029	0.069	0.182
PC → ACS → EE	0.110	0.019	0.075	0.146
PC → EFE → ACS → EE	0.024	0.009	0.009	0.044
Direct effects: PC → EE	0.471	0.039	0.395	0.546
Total indirect effect	0.258	0.042	0.176	0.335
Total effect	0.729	0.037	0.656	0.802

PC, Psychological Capital; EE, Employment expectations; EFE, Educational flow experience; ACS, Active coping style.

## 4 Discussion

### 4.1 The impact of psychological capital on employment expectations

This study explored the influence of psychological capital on the employment expectations of vocational undergraduate students and found a significant positive predictive effect, supporting Hypothesis 1. In today's complex and uncertain higher education and employment landscape, understanding how individuals construct a stable and positive career identity has become a pressing issue (James et al., 2021). Psychological capital, reflecting the integration of internal resources and adaptive capacity, plays a fundamental role in shaping career beliefs, motivational systems, and goal-directed behavior (Zhou W. et al., 2024).

Psychological capital contributes not only to emotional regulation and self-worth maintenance but also translates into problem-solving ability, persistence, and future orientation in career contexts (Belle et al., 2022). Rather than viewing psychological capital as a single predictor, it should be understood as an embedded resource that influences multiple stages of career development, from motivation initiation to identity integration. Given the transformation of vocational education systems, educational interventions that foster psychological capital may be critical to enhancing students' long-term adaptability and development potential.

### 4.2 Chain mediation of educational flow and active coping

The study further revealed a significant chain mediation effect through the path “educational flow → active coping,” supporting Hypothesis 4. This finding is largely consistent with previous research (Georgiou et al., 2021) and offers new theoretical insight into how psychological capital operates through complex psychosocial mechanisms to shape employment cognition.

Specifically, the chain mediation model uncovered the following psychological pathway: first, psychological capital enhances students' positive perceptions of educational equity, upward mobility, and returns to education, thereby improving their educational flow experience (Kawalya et al., 2019). Educational flow, reflecting cognitively enriched beliefs about the empowering nature of education, helps students develop a clear and optimistic outlook on future opportunities, motivating them to adopt active coping strategies.

Second, educational flow positively predicts active coping (Khedmatian et al., 2022). When students perceive education as a viable pathway for social mobility, they are more likely to address challenges using problem-focused strategies. This conversion from institutional trust to behavioral motivation highlights how educational flow mobilizes students' psychological resources, enabling them to exhibit resilience and goal orientation in uncertain job markets.

Such coping strategies, in turn, enhance students' sense of control and sustained effort toward employment goals, indirectly reinforcing employment expectations. Participants showed greater self-efficacy, problem-solving capacity, and perseverance, shaping more optimistic and defined career expectations (Belle et al., 2022). This chain mediation pathway demonstrates that psychological capital influences employment expectations not only directly but also indirectly via a cognitive-affective-behavioral sequence.

Importantly, the chain model underscores the indirect and progressive nature of psychological capital's effect. Compared with single mediation paths, this sequential model better reflects the complexity of real-life psychological processes (Hou and Hu, 2024) and highlights the layered transmission of internal resources across beliefs, strategies, and outcomes. This finding also emphasizes that psychological capital development and educational flow experience should be jointly addressed to foster students' employment readiness.

### 4.3 Independent mediation and suppressor effects

Further analyses confirmed that educational flow and active coping independently mediated the relationship between psychological capital and employment expectations, supporting Hypotheses 2 and 3, which is consistent with previous studies (Adil et al., 2020; Sun et al., 2022). However, both mediators exhibited suppressor effects, meaning they attenuated the direct effect of psychological capital.

A suppressor effect occurs when introducing mediators weakens or reverses the direct relationship between the independent and dependent variables (MacKinnon et al., 2000). In this study, introducing educational flow and active coping reduced the strength of the direct path from psychological capital to employment expectations. This suggests that the mediators may partially obscure or counterbalance the direct positive influence of psychological capital.

In the case of educational flow, while high psychological capital typically enhances students' perceived control and future orientation, their perceptions of fairness and opportunity within the education system may be influenced by external instability. Students with high psychological capital but low perceived educational equity may moderate their expectations due to perceived systemic barriers (Sesen and Ertan, 2020), leading to a negative suppressor effect.

For active coping, although it is generally a beneficial resource, in highly competitive or structurally unequal employment environments, excessive reliance on individual coping may result in internalizing all responsibility for job outcomes. This overemphasis on self-responsibility can trigger stress and anxiety (Hecht, 2013), particularly when external limitations are ignored, thereby reducing employment expectations.

In summary, while both educational flow and active coping served as mediators, they also partially suppressed the direct effect of psychological capital on employment expectations. These findings deepen our understanding of mediation complexity and highlight the need to consider dual-function effects of seemingly positive variables in psychological models.

## 5 Conclusion

This study systematically investigated the relationship between psychological capital and employment expectations among vocational undergraduate students and verified the mediating roles of educational flow and active coping. Results revealed that psychological capital significantly predicts employment expectations, both directly and indirectly through two mediators, which also form a significant chain pathway.

Notably, the analysis uncovered suppressor effects, wherein the mediators weakened the direct influence of psychological capital. This finding challenges the conventional assumption that mediators uniformly enhance effects and highlights the potential for unintended cognitive and behavioral side effects.

Theoretically, the identified chain mediation model—“psychological capital → educational flow → active coping → employment expectations”—offers a nuanced framework for understanding how internal psychological resources shape career-related outcomes via cognitive and behavioral channels. The suppressor effect further refines our understanding by illustrating the complexity and context-dependence of positive psychological mechanisms.

Practically, the findings suggest that targeted interventions are needed to cultivate psychological capital among vocational students, such as training programs to enhance self-efficacy, hope, optimism, and resilience (Martono et al., 2022). Simultaneously, educational institutions should foster immersive and empowering learning environments to improve students' perceptions of educational mobility. However, educators and policymakers must also avoid overemphasizing personal responsibility in coping strategies, especially in structurally constrained job markets, as this may inadvertently lower students' career optimism.

This study has several limitations. (1) The sample was drawn from a single vocational university in Guangdong Province, which may limit generalizability. Future research should examine broader and more diverse populations. (2) This study focused on three key variables—psychological capital, educational flow, and active coping—while other potential influences on employment expectations, such as family capital or personality traits, were not included. Future studies should consider these additional factors to provide a more comprehensive understanding.

Future research should consider longitudinal designs to explore the temporal dynamics of suppressor effects and examine contextual moderators, such as perceived policy fairness or labor market signals, that shape the translation of psychological resources into career attitudes. Further exploration of the interplay between psychological traits, educational experiences, and coping behaviors can provide a more holistic basis for supporting the career development and adaptability of vocational undergraduates.

## Data Availability

The datasets presented in this study can be found in online repositories. The names of the repository/repositories and accession number(s) can be found in the article/supplementary material.
